# Combination of UV and green light synergistically enhances the attractiveness of light to green stink bugs *Nezara* spp

**DOI:** 10.1038/s41598-022-16295-z

**Published:** 2022-07-19

**Authors:** Nobuyuki Endo, Mantaro Hironaka, Yoshiyuki Honda, Tetsuhiro Iwamoto

**Affiliations:** 1 Institute for Plant Protection, National Agriculture and Food Research Organization (NARO), Tsukuba, Ibaraki 305-8666 Japan; 2 Department of Bioproduction Science, Ishikawa Prefectural University, Nonoichi, Ishikawa 921-8836 Japan; 3 Yamaguchi Prefectural Agriculture and Forestry General Technology Center, Yamaguchi, Yamaguchi 753-0231 Japan

**Keywords:** Entomology, Behavioural ecology, Conservation biology

## Abstract

The southern green stink bug *Nezara viridula* and its congener *N. antennata* are important agricultural pests worldwide. These species show positive phototaxis and their compound eyes have high sensitivity to UV and green lights. The attractiveness of monochromatic UV, green lights and combined UV and green light to stink bugs was investigated under field conditions. The number of stink bugs caught increased with the number of UV LEDs, but very few bugs were caught using green light, irrespective of the number of LEDs. However, the most stink bugs were caught when both colors were combined. These results indicate that monochromatic green light is less attractive to *Nezara* bugs, but when mixed with UV light, it synergistically enhances the attractiveness of UV light. This finding contributes to the construction of reliable and highly specific light traps to monitor *Nezara* bugs. The addition of green light hardly affected the attractiveness of the UV light to other insects, such as *Anomala* beetles, which are often caught in light traps. We conclude that the spectral composition of light that is attractive to nocturnal insects depends on the species, hence it is possible to make ecologically friendly light traps that are target specific.

## Introduction

The southern green stink bug *Nezara viridula* (L.) (Heteroptera: Pentatomidae) is a cosmopolitan pest distributed from tropical to temperate regions in America, Africa, Asia, Australia, and Europe, with significant economic impacts on various crops worldwide^1,2^. Over the last 20 years, its range has expanded northward in Japan, possibly due to global warming^3–5^, becoming a major soybean pest in these areas^6^. Around the northern limit of its distribution, *N. viridula* coexists with a congeneric species, the oriental green stink bug *Nezara antennata* Scott^3–5^, which is widely spread throughout Japan^7^. In these sympatric distributions, *N. viridula* often replaces *N. antennata*^4,8–10^.

Automatic daily monitoring of pest insects, such as stink bugs and plant hoppers, is now conducted using light traps throughout Japan^11–14^. *Nezara* bugs exhibit positive phototaxis, and thus light traps are useful for monitoring these populations^15^. However, conventional light traps are too large and heavy to carry around and require a fixed power supply. To develop more convenient light traps, we must find visual stimuli with high attraction efficiency, even at low brightness, and at low power consumption.

Light has several properties, among which the composition of wavelength and light intensity seem to be the most important factors affecting its attractiveness to insects^16^. In behavioral bioassays, *N. viridula* adults strongly prefer ultraviolet (UV) light over longer-wavelength light^17^. Thus, UV light is considered to be the most suitable for light traps. The compound eyes of *N. viridula* adults show strong sensitivity to UV regions and also green regions in electroretinograms (ERG)^17^; suggesting the potential for behavioral responses or biological roles of green light.

There have been several reports on the attractiveness of monochromatic light to insects, but only a few have investigated the attractiveness of multichromatic light. The greenhouse whitefly *Trialeurodes vaporariorum* Westwood is highly attracted to green light and less to UV light^18,19^, but the addition of UV light enhances the attractiveness of green light^19^. This suggests the addition of green light, which shows a strong ERG response, might improve the attractiveness of UV light to *Nezara* bugs.

In this study, we evaluated the attractiveness of mono- and multichromatic UV and green lights under field conditions to develop an effective light trap for *Nezara* bugs. We also evaluated the attractiveness of mono- and multichromatic lights to other stink bugs and beetles that are often caught in light traps. Our study reports novel finding that green light synergistically enhanced the attractiveness of UV light to some stink bugs, including *Nezara* spp, and provides insights into developing environmentally friendly species-specific traps.

## Results

### Attractiveness of light sources to *Nezara* species

**Figure 1:**
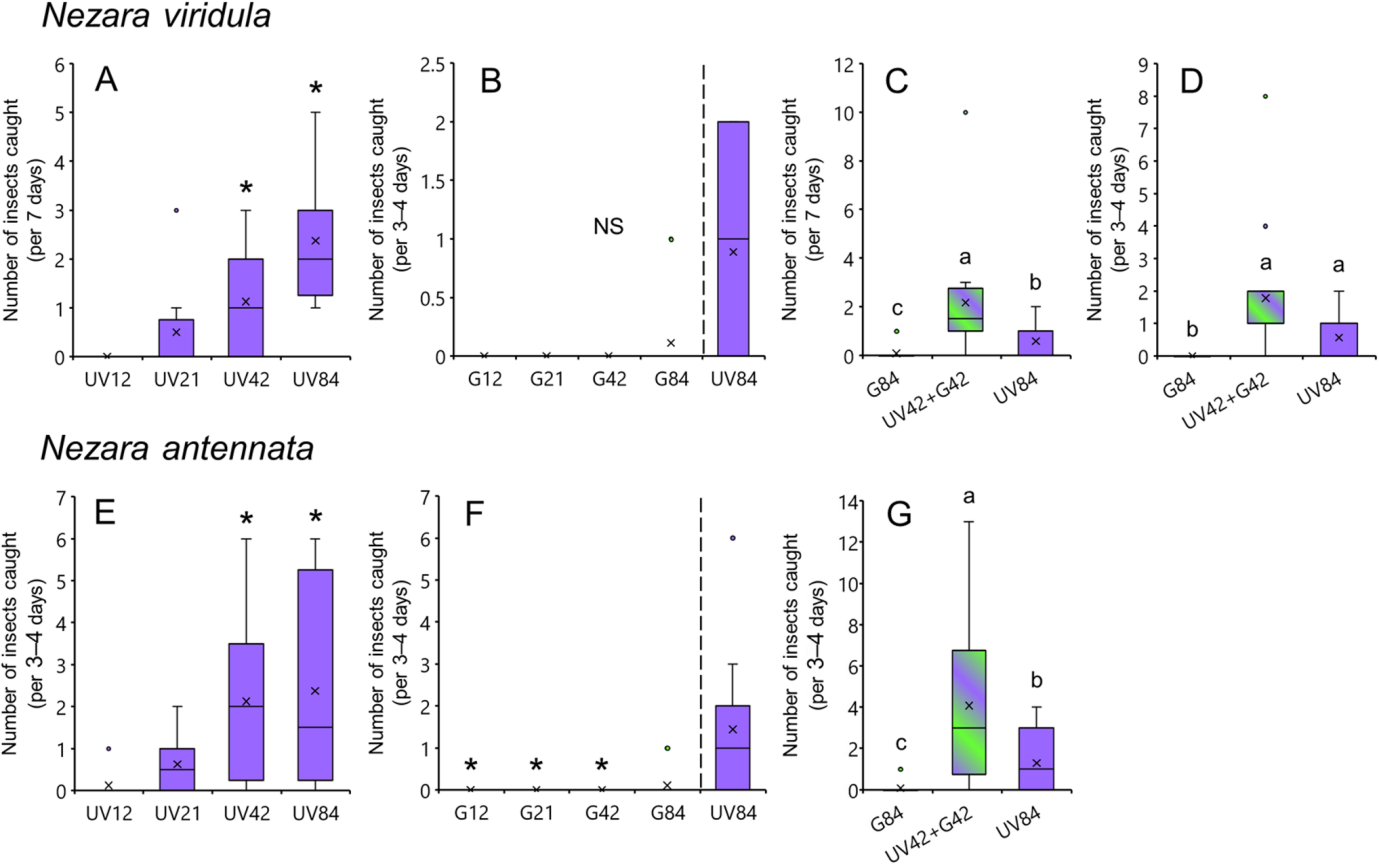
Attractiveness of different light sources to *Nezara viridula* (top) and *N. antennata* (bottom). The graphs on the left present the attractiveness of UV light at different intensities, the middle graphs present the attractiveness of green light at different intensities, and the graphs on the right present the attractiveness of the combined UV and green light. The number after UV or G indicates the number of LEDs used for the light source. Boxplots represent the median value (horizontal line), mean value (cross mark), interquartile range (boxed area), maximum and minimum values (vertical bar), and outlier value (circle). Asterisks indicate significant differences (*p* < 0.05) between 12 UV-LEDs in graphs A and E (Shirley–Williams test), and between 84 UV-LEDs in graph F (Wilcoxon signed-rank test). Different letters in the same graph indicate significant differences between light sources (Wilcoxon signed-rank test with Bonferroni correction; *p* < 0.05 in graphs C, D, and G). *NS* not significant (*p* > 0.05). The data in graphs A and C were obtained from field experiments conducted in Okinawa, data in graphs B, D, F, and G were collected in Yamaguchi, and data in graph E were collected in Niigata. Due to time constraints, the insects captured in traps were counted every 7 days in Okinawa, and every 3–4 days in Niigata and Yamaguchi.

*Nezara* spp. showed a light intensity-dependent reaction to UV light but were less attracted to green light. They also showed a reaction to the synergistic combination of these two colors. The number of *Nezara* bugs caught tended to increase as the number of UV-LEDs increased (Fig. 1A,E). Traps with 42 and 84 UV-LEDs caught significantly more *Nezara* bugs than those with 12 UV-LEDs (Shirley–Williams test; *p* < 0.05). Few bugs were caught in the green light traps, irrespective of the number of LEDs and there were no significant differences between the green lights (Shirley–Williams test; *p* > 0.05), while many stink bugs were caught in UV light traps (Fig. 1B,F). Traps with alternating 42 UV- and 42 green-LEDs caught significantly more stink bugs than traps with only 84 UV- or 84 green-LEDs (Wilcoxon signed-rank test with Bonferroni correction; *p* < 0.05) (Fig. 1C,G). A similar result was obtained for *N. viridula* in another field experiment conducted in Yamaguchi (Fig. 1D).

### Attractiveness of combined light sources to other stink bug species

**Figure 2:**
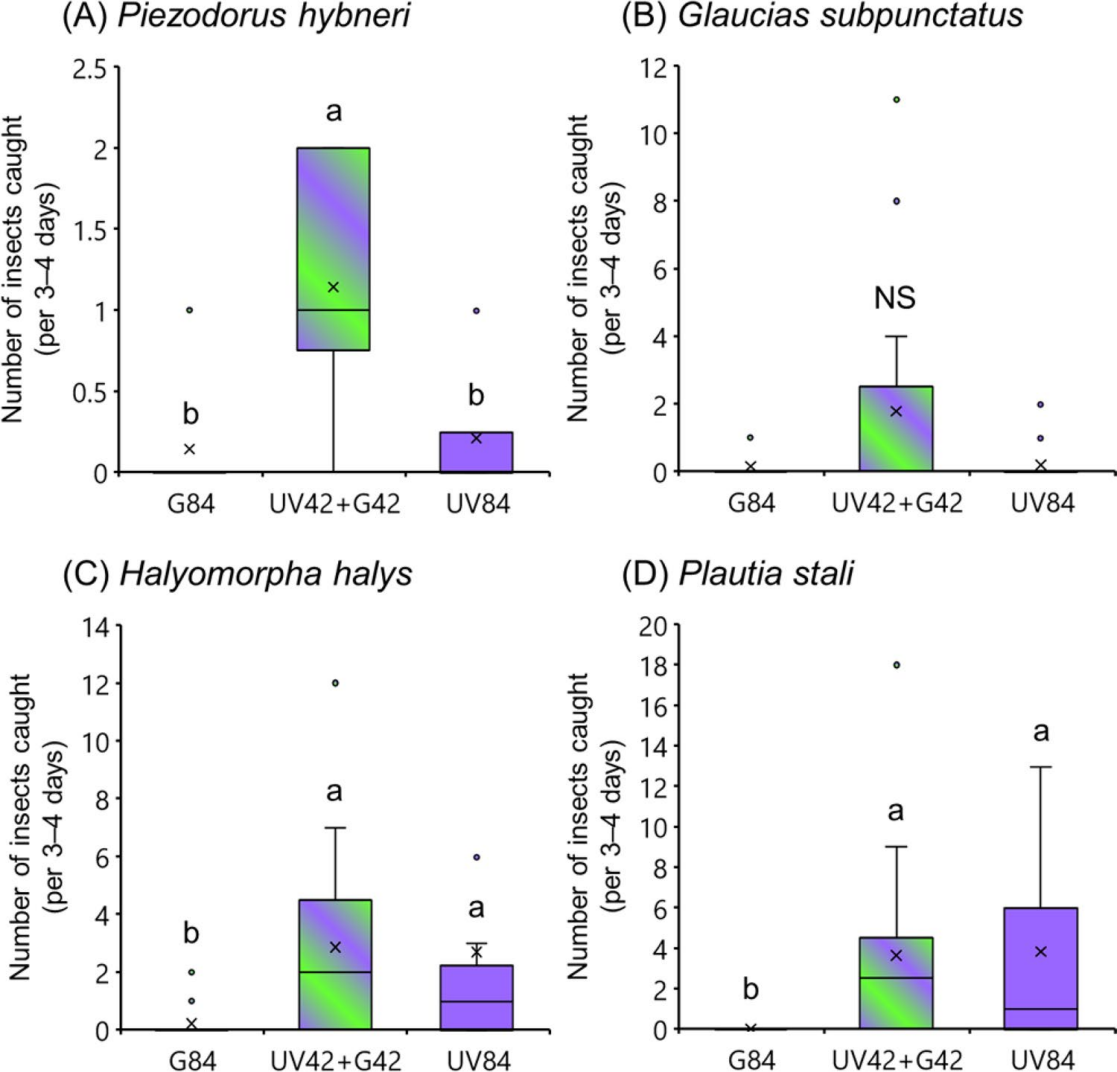
Attractiveness of different light sources to stink bugs. Boxplots represent the median value (horizontal line), mean value (cross mark), interquartile range (boxed area), maximum and minimum values (vertical bar), and outlier value (circle). Different letters above the bars indicate significant differences (*p* < 0.05) in Wilcoxon signed-rank test with Bonferroni correction. *NS* not significant (*p* > 0.05). The data shown in these graphs were all obtained from field experiments conducted in Yamaguchi.

More than five times as many *Piezodorus hybneri* (Wilcoxon signed-rank test with Bonferroni correction; *p* < 0.05) and eight times as many *Glaucias subpunctatus* (but not significantly more, *p* > 0.05) were caught in the combined color light trap than the monochromatic UV light trap (Fig. 2A,B). Other stink bug species, *Halyomorpha halys* and *Plautia stali,* were caught in equal numbers in the combined color and monochromatic UV light traps (Fig. 2C,D).

### Attractiveness of combined light sources to beetle species

**Figure 3:**
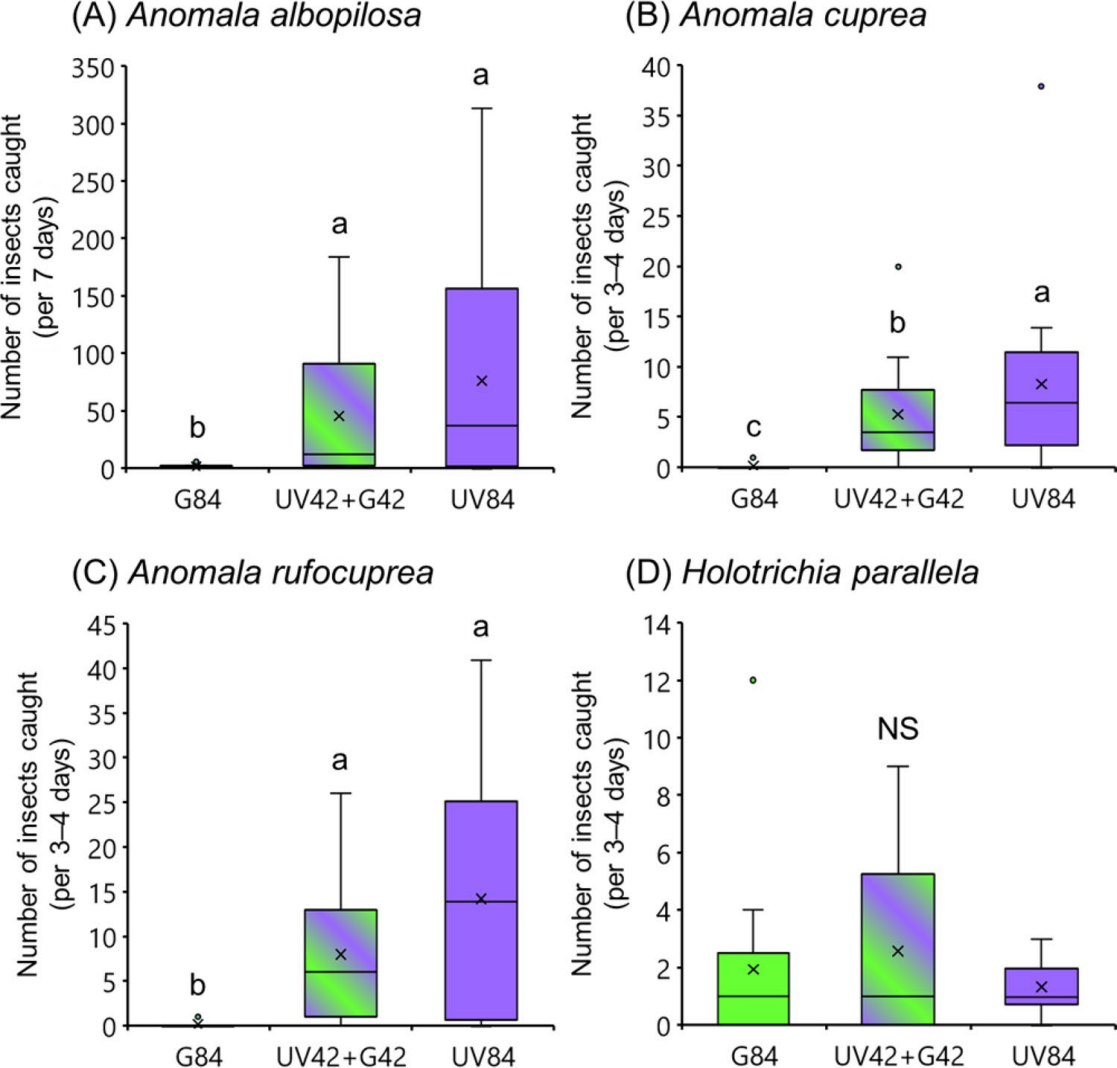
Attractiveness of different light sources to beetles. Boxplots represent the median value (horizontal line), mean value (cross mark), interquartile range (boxed area), maximum and minimum values (vertical bar), and outlier value (circle). Different letters above the bars indicate significant differences (*p* < 0.05) in Wilcoxon signed-rank test with Bonferroni correction. *NS* not significant (*p* > 0.05). The data shown in these graphs were obtained from field experiments conducted in Yamaguchi, except for *A. albopilosa* which was conducted in Okinawa.

The difference in attractiveness between the three light sources was almost the same in the three *Anomala* beetles (Fig. 3A–C). Only a few *Anomala* beetles were caught in the green light traps, while significantly more beetles were caught in the UV and combined color light traps (Wilcoxon signed-rank test with Bonferroni correction; *p* < 0.05). *Holotrichia parallela* was caught in traps with monochromatic green and UV lights (Fig. 3D). Most *H. parallela* were caught in the trap with combined UV and green light, but there was no significant difference in the numbers caught among the light traps (Friedman test: χ^2^ = 0.1739, df = 2, *p* = 0.9167).

## Discussion

Our field bioassays with different light traps demonstrated that monochromatic green light was less attractive for *Nezara* bugs, but when combined with UV light, it synergistically enhanced the attractiveness of UV light. There have been several reports of the attractiveness of monochromatic light to insects, but only a few have investigated the attractiveness of combinations of light colors. Kirkpatrick et al.^20^ compared the attractiveness of green and UV light alone and in combination to stored product insects but did not find any significant differences between either of the monochromatic light and the combined color light. The greenhouse whitefly *T. vaporariorum* is attracted to monochromatic green light, which is increased when combined with UV light^19^. Similarly, the Asian citrus psyllid, *Diaphorina citri*, is strongly attracted to UV combined with yellow or green light^21^. In contrast, blue light suppresses the attractiveness of green light to whitefly^22^. Thus, the responses to combinations of colored lights differ depending on the species or wavelength composition. To the best of our knowledge, this is the first report to demonstrate that the combination of different wavelengths of light synergistically enhanced trap catches of nocturnal insects. In addition, these results were obtained from field evaluations, where combinations of UV and green light can be readily used for practical applications.

The compound eyes of insects have several photoreceptor cells with different spectral sensitivities, and each photoreceptor cell perceives a specific range of wavelengths. In *N. viridula*, there seem to be three types of photoreceptor cells that are sensitive to UV, blue, and green light^17^; thus, the UV and green lights used in our experiments could be perceived as different stimuli. Why are *Nezara* bugs strongly attracted to a combination of UV and green light? One possible explanation is that stimuli with two different wavelengths that cause different output behaviors might improve the capture efficiency. Different wavelengths are known to induce different “wavelength-specific behaviors” in some diurnal hemipteran insects. UV radiation elicits behavior involved in flight initiation, migration, and dispersal^22–24^, while green-yellow light induces settling on host plants^22–25^. *T. vaporariorum* prefers illumination of these two different lights (UV and green) together^19^. Even in nocturnal stink bugs, different behaviors might be elicited by light of different wavelengths. Stink bugs often pass next to a UV light source and fewer bugs land on or hit a UV light source^16^. Although UV alone is highly attractive to *Nezara* bugs, it seems that the accuracy of orientation to the stimulus is not necessarily high. If green light elicits orientation or landing in these bugs, which often feed on young seeds of green plants such as soybeans and rice, the green light could improve the orientation accuracy. In addition, green light may enhance downward flight and promote landing, which lead the bugs into the trap chamber placed under the light source. The wavelength-specific behavior of nocturnal insects has not been well documented. The validity of this hypothesis could be clarified by observing the orientation position for each wavelength light or the process of capture in the trap in more detail.

Another possibility is that the simultaneous input of the two wavelengths made the light source of the trap more attractive to the insects as an orientation target. Previous studies have shown that vision plays an important role in the stable orientation of nocturnal insects^26^. Flying insects in particular need to accurately orient to objects such as flowers, leaves, and branches, either to land or to avoid during flight. To do this, they must be able to distinguish between the sky and objects by using the faint light of the moon or night sky. Wehner^27^ suggested that insect sensitivity to both UV and blue-green wavelengths is related to wide-field motion detection, that is, orientation and navigation using the contrast between the UV-rich sky and the UV-unreflective earth. Similarly, Möller^28^ suggested that insects can use UV-green or UV-blue contrasts to distinguish between celestial and terrestrial objects. In this study, the UV and green LEDs were placed approximately several centimeters apart, so that the insects could perceive the light source as a space where the sky and objects had greater contrast and were easier to distinguish from the surroundings. It is possible that the insects were strongly attracted to the light source with two wavelengths as a more reliable and stable flight orientation.

Although green light enhanced the attractiveness of UV light to stink bugs, the degree of attractiveness differed greatly, even in the stink bug subfamily (Pentatomidae: Pentatominae). The color combination was synergistic and strong for *P. hybneri* and *G. subpunctatus* as well as *Nezara* species, but additive and weak for *H. halys* and *P. stali*. This indicated that UV light dependency, and the roles or the degree of contribution of other light differed among stink bug species. The addition of green light hardly affected the attractiveness of the UV light to *Anomala* beetles, whereas monochromatic green light was attractive and additively enhanced the attractiveness of the UV light to *H. parallela*. These results suggest that strong attractiveness to a combination of UV and green light is not common in nocturnal insects and depends on the species. In addition, the compound eyes of *Anomala* beetles have photoreceptors that are sensitive to green, blue, and UV and show similar spectral sensitivity curves^29^ to *N. viridula*^17^. Therefore, the reactivity to combined light does not correspond to the known spectral sensitivity curves of these insects.

Overall, these results can contribute to making reliable, highly accurate monitoring traps for *Nezara* stink bugs. The results also show that the light intensity or the number of LEDs can be reduced while maintaining a threshold of attractiveness, saving power and costs. In addition, since many nocturnal insects are attracted to artificial light^30,31^, ecological disturbance caused by light has become a serious problem^32–34^. Even in light traps, many non-target species are caught and killed, which complicates sorting. Therefore, it is preferable to use a light source that is target specific. Taken together, our findings can contribute to the development of both economically and environmentally friendly monitoring light traps for stink bug species, including *Nezara* bugs.

## Methods

### LED traps

**Figure 4:**
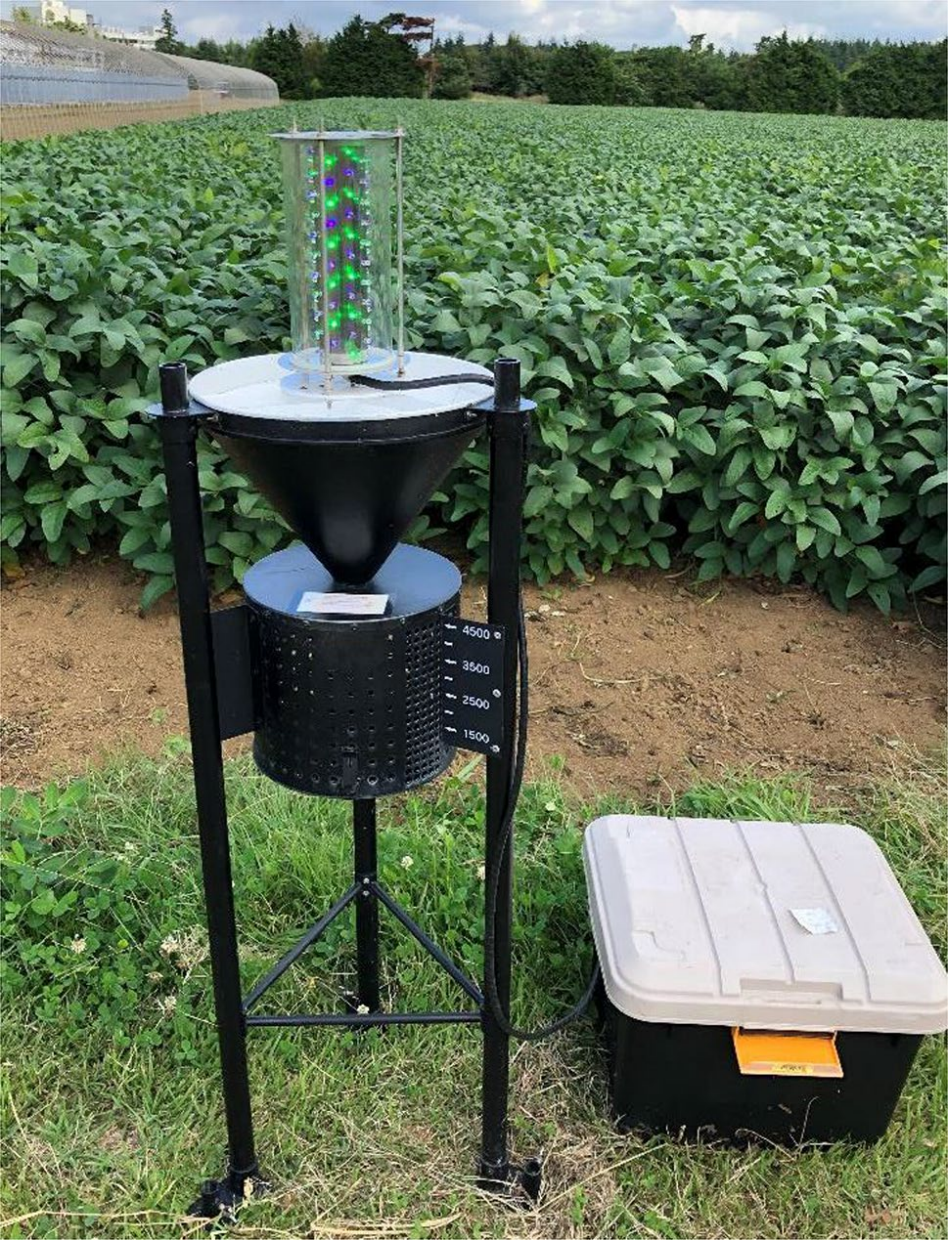
Photograph of combined UV and green LED trap used in the experiments.

We used a commercially available portable light trap (Eco-chu trap, Konan Shisetsu Kanri, Okinawa, Japan) to modify the light source. A prototype trap equipped with 12 UV-LED bulbs was developed to catch the green chafer *Anomala albopilosa* (Hope)^35^, but it was not sufficiently attractive to stink bugs. Light sources with different numbers of LEDs, from 12 to 84, were used. Either or both bullet-type UV-LED bulbs (NS395L-ERLO; 395 nm, 20 mA, Nitride Semiconductors, Tokushima, Japan) and green LED bulbs (NEPG510S; 525 nm, 20 mA, Nichia, Tokushima, Japan) were used. LEDs were arranged vertically on a stainless-steel cylinder (4.8 cm in diameter, 20 cm in height). Light sources with 12 LEDs were arranged in six rows around the circumference. Each row was arranged as two LEDs at 7.8 cm intervals. Adjacent LEDs were arranged in a left-handed spiral (depression angle of 53°, at approximately 2.5 cm intervals). Light sources with 21 LEDs were arranged in eight rows around the circumference. Each row was arranged as two or three LEDs at 7.2 cm intervals. Adjacent LEDs were arranged in a left-handed spiral (depression angle of 63°, at approximately 2.0 cm intervals). Light sources with 42 LEDs were arranged in eight rows around the circumference. Each row was arranged as five or six LEDs at 3.6 cm intervals. Adjacent LEDs were arranged in a left-handed spiral (elevation angle 63°, at approximately 2.0 cm intervals). Light sources with 84 LEDs were arranged in eight rows around the circumference. Each row was arranged as 10 or 11 LEDs at 1.8 cm intervals. Adjacent LEDs were arranged in a left-handed spiral (elevation angle 63°, at approximately 2.0 cm interval). When both UV and green LEDs were used, both LEDs were arranged alternately in a row (Fig. 4). The cylinder with the LEDs was covered with a transparent acrylic cylinder (9.8 cm in diameter, 20 cm in height).

The light source was mounted on a funnel (31 cm in diameter, 24 cm in height), and the lower part of the light source was approximately 100 cm above the ground. A cylindrical chamber (23 cm in diameter, 20 cm in height) was placed under the funnel so that insects that were attracted to the light fell into the funnel and were trapped. The legs of the trap were anchored to the ground using steel stakes. A dimethyl-dichloro-vinyl-phosphate (DDVP) plate containing 10.7 g dichlorvos (Bapona, Earth Chemical, Tokyo, Japan) was placed inside the chamber to kill the insects. The lights were turned on at 18:00 and turned off at 6:00 the next day. The power for the lights was supplied by rechargeable car batteries (N-40B19R/SB; DC 12 V, 28 Ah, Panasonic, Osaka, Japan) or domestic electricity power supplies (AC100V).

### Emission spectra of combined UV and green light

**Figure 5:**
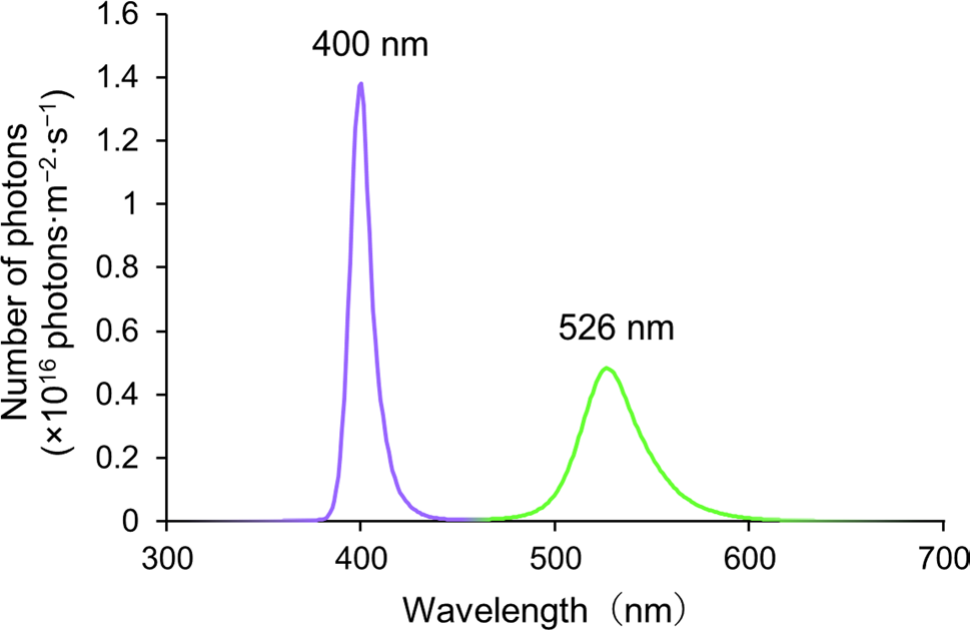
Emission spectra of light source with UV- and green-LEDs. The light source was composed of alternating 42 UV-LEDs and 42 green-LEDs. The intensity of light was measured using a high-speed spectrometer (HSU-100S). An attached sensor fiber was placed 50 cm in front of the light source.

The spectral intensity of combined UV and green light was measured using a high-speed spectrometer (HSU-100S, Asahi Spectra, Tokyo, Japan) in a dark room. An attached sensor fiber was placed 50 cm in front of the light source. The measurement was performed five times, the light source was rotated for each measurement to minimize the angle effect, and the average was used as a representative value. The UV- and green-LED emission spectra showed single peaks at wavelengths of 400 and 526 nm, respectively (Fig. 5). Calculated light intensities of UV (350–450 nm) and green (451–600 nm) regions were 2.12 × 10^17^ and 2.03 × 10^17^ photons m^−2^ s^−1^, respectively; that is, the light intensities of UV- and green-LEDs were almost equal.

### Field evaluation of attractiveness to light sources

Field experiments were conducted at three locations in Japan: Central Region Agricultural Research Center (CARC), Hokuriku Research Station (37° 07′ 00″ N, 138° 16′ 23″ E) in Niigata; Yamaguchi Prefectural Agriculture & Forestry General Technology Center (YPATC) (34° 09′ 37″ N, 131° 29′ 47″ E) in Yamaguchi; and Okinawa Prefectural Agricultural Research Center (OPARC) (26° 06′ 18″ N, 127° 40′ 53″ E) in Okinawa. The distribution of *Nezara* spp. varies among the regions in Japan. Only *N. antennata* is distributed in Niigata, and only *N. viridula* is distributed in Okinawa. Both *N. antennata* and *N. viridula* were found in Yamaguchi.

### Experiment 1: Attractiveness of UV light at different intensities

Field experiments to evaluate the attractiveness of UV light at different intensities were conducted from August 2 to 29, 2017, around a soybean field at the CARC in Niigata and from July 12 to September 9, 2019, in grassland at the OPARC in Okinawa. Light traps with different numbers of UV-LEDs (12, 21, 42, and 84) were used as light sources. Each of the four LED traps was spaced more than 30 m apart and placed randomly around the soybean field or grassland. Due to time constraints, the numbers of *N. viridula and N. antennata* captured in traps were counted every 3–4 days at Niigata (total eight replicates) and every 7 days in Okinawa (total eight replicates). The traps were randomly repositioned every week to minimize the effect of trap location. The raw capture data for each trap are listed in Supplementary Table S1.

### Experiment 2: Attractiveness of green light at different intensities

Field experiment to evaluate the attractiveness of green light at different intensities was conducted from July 5 to August 5, 2019, around a soybean field at the YPATC in Yamaguchi. Light traps with different numbers of green LEDs (12, 21, 42, and 84) were used as light sources. Light trap with 84 UV-LEDs was used as the positive control. Each of the five LED traps was spaced more than 30 m apart and placed randomly around the soybean field. The numbers of *Nezara* bugs captured in traps were counted every 3–4 days (total nine replicates). The traps were randomly repositioned every week. The raw capture data for each trap are listed in Supplementary Table S2.

### Experiment 3: Attractiveness of combined-UV and green light

Field experiments to evaluate the attractiveness of combinations of UV- and green-LEDs were conducted from June 13 to September 4, 2017, in the grassland at the OPARC in Okinawa, and from July 15 to September 1, 2017, around a soybean field at the YPATC in Yamaguchi. Light traps with 84 UV-LEDs, 84 green-LEDs, and a combination of 42 UV-LEDs and 42 green-LEDs were used as light sources. Each of the three LED traps was spaced more than 30 m apart and placed randomly around the soybean field or grassland. Although insects other than *Nezara* bugs (mainly coleopteran species) were captured in the light traps, for soybean pests, the funnel-type light traps are intended for monitoring large coleopteran and heteropteran insects (> 1 cm). Therefore, we targeted and counted insects that meet these conditions. Statistical analysis was performed on species with a total capture number of more than 20 individuals in the three traps. The species were as follows: in addition to *Nezara* bugs, heteropteran bugs, *Piezodorus hybneri* (Gmelin), *Glaucias subpunctatus* (Walker), *Halyomorpha halys* (Stål), and *Plautia stali* Scott, as well as coleopteran beetles, *Anomala albopilosa* (Hope), *A. cuprea* Hope, *A. rufocuprea* Motschulsky, and *Holotrichia parallela* Motschulsky. The insects captured in traps were counted for each species every 7 days at Okinawa (total 12 replicates) and every 3–4 days at Yamaguchi (total 14 replicates). The traps were randomly repositioned every week. The raw capture data for each trap are listed in Supplementary Table S3.

### Data analysis

In Experiment 1, the effect of UV light intensities for trap catches were analyzed using a nonparametric one-tailed Shirley–Williams test under an assumption that higher light intensity attracts larger amounts of insects. In Experiment 2, the effect of green light intensities for trap catches were analyzed using the Shirley–Williams test. Subsequently, the attractiveness of each green light was compared to that of UV light using Wilcoxon matched pairs signed-rank test. In Experiment 3, the effect of light sources for trap catches was analyzed using the Friedman test, followed by the Wilcoxon signed-rank test, with Bonferroni correction for multiple comparisons. Statistical analyses were performed using R version 4.2.0 (R Core Team, 2022).

## Supplementary Information

Supplementary Information.

## Data Availability

All data that support the findings of this study are provide in the manuscript and supplementary file or are available from the corresponding author on reasonable request.
